# Sexual dysfunction associated with 5α-reductase inhibitors in the treatment of androgenetic alopecia: a systematic review

**DOI:** 10.3389/fmed.2026.1787706

**Published:** 2026-06-18

**Authors:** Aleksandra Złotowska, Beata Jastrząb-Miśkiewicz, Piotr K. Krajewski

**Affiliations:** 1 University Centre of General Dermatology and Oncodermatology, Wrocław Medical University, Wrocław, Poland; 2 Division of Dermatology, Venereology and Clinical Immunology, Faculty of Medicine, Wrocław University of Science and Technology, Wrocław, Poland; 3 Department of Dermatovenereology, 4th Military Hospital, Wrocław, Poland; 4 Lübeck Institute of Experimental Dermatology, University of Lübeck, Lübeck, Germany

**Keywords:** 5α-reductase inhibitors, androgenetic alopecia, dutasteride, finasteride, sexual dysfunction, sexual side effects

## Abstract

**Introduction:**

5α-reductase inhibitors (5-ARIs) are most commonly used to treat benign prostatic hyperplasia (BPH) and androgenetic alopecia (AGA). Preclinical and clinical studies suggest that in patients with AGA treated with 5-ARIs, a subset reports sexual adverse events, including decreased libido, erectile dysfunction, and ejaculatory disorders. This review explores the development of sexual dysfunction in patients with androgenetic alopecia due to the use of 5-ARIs, emphasizing their importance in the clinical diagnosis of health disorders coexisting with hair loss.

**Methods:**

A systematic review was conducted by searching electronic databases, including MEDLINE, Scopus, Web of Science and Google Scholar, according to the PRISMA guidelines. The search was limited to articles published in English and up to December 2025. Key search terms included “5α-reductase inhibitors” or “5-ARIs” or “finasteride” or “dutasteride” AND “side effects” or “sexual side effects” or “sexual” or “sexual dysfunction” AND “androgenetic alopecia” or “male pattern hair loss” or “female pattern hair loss.” Data synthesis included findings from 41 studies, comprising 33 primary-evidence studies in AGA/MPHL/FPHL populations and 8 supporting-evidence studies providing pharmacokinetic, mixed-indication, or comparative context.

**Results:**

5-ARIs are effective therapies for androgenetic alopecia and are generally well tolerated. Across placebo-controlled RCTs evaluating oral finasteride 1 mg in men with AGA, sexual adverse events were reported in 1.9–6.7% of treated patients compared with 0.9–3.9% in placebo groups, with most events mild and reversible upon discontinuation. Topical finasteride 0.25% was associated with lower rates of sexual adverse events (2.8%) compared with oral finasteride (4.8%). For dutasteride 0.5 mg, sexual adverse events ranged from 4.1 to 12.0% across RCTs, compared with 4.0–5.0% in placebo groups. No sexual adverse effects were consistently reported in women treated with 5-ARIs for AGA. In most reports, the effects were transient and reversible after discontinuation.

**Conclusion:**

Overall, clinicians should counsel patients that most sexual side effects reported in controlled AGA studies are infrequent, mild, and reversible, but individual susceptibility varies. Shared decision-making, careful monitoring of sexual function, and further high-quality long-term studies—using standardized definitions of sexual dysfunction and persistence—are needed to quantify risks better and identify vulnerable subgroups.

## 1 Introduction

Androgenetic alopecia (AGA) is a non-scarring hair loss characterized by progressive follicular miniaturization and characteristic patterned alopecia (1). It is the most common cause of hair loss in adults. It is estimated to affect approximately 80% of men and 50% of women by the age of 70, with its prevalence increasing with age (2). In men, gradual thinning of the hair is visible in the vertex and frontal-temporal areas (3). Clinically, men typically show frontotemporal recession and vertex thinning, whereas women more often present with diffuse central scalp thinning with relative preservation of the frontal hairline (4, 5). The distribution reflects regional differences in androgen sensitivity and androgen receptor expression across the scalp (4, 5). The pathogenesis of AGA is determined by androgen-dependent follicular miniaturization in genetically predisposed individuals. Potential contributors include increased local DHT signaling (e.g., via 5α-reductase activity), altered androgen receptor signaling, and individual genetic susceptibility. Moreover, disorders of androgen metabolism led to a gradual shortening of the anagen phase of hair growth and to progressive miniaturization of hair follicles, resulting in hair loss (3). In addition, the telogen phase is prolonged, leading to visible thinning (6).

5α-reductase inhibitors (5-ARIs), including finasteride and dutasteride, inhibit the 5α-reductase enzyme, thereby reducing the conversion of testosterone to DHT and downstream androgen signaling in androgen-sensitive tissues such as the prostate and scalp hair follicles (7). 5-ARIs are most commonly used to treat benign prostatic hyperplasia (BPH) and androgenetic alopecia (AGA) (8). Preclinical and clinical studies indicate the use of 5-ARIs in patients with AGA has been associated with sexual adverse events in a subset of individuals, including decreased libido, erectile dysfunction, and ejaculatory disorders (9, 10).

The World Health Organization (WHO) defines sexual health as “a state of physical, emotional, mental and social wellbeing in relation to sexuality; it is not merely the absence of disease, dysfunction or infirmity” (11). Sexual health is an integral part of physical and mental health. It is recognized as an inherent factor in wellbeing that significantly impacts adaptation during difficult life stages, including chronic diseases (12). Sexual dysfunction can occur as an undesirable side effect of taking many medications. Drug-related sexual adverse effects may include decreased libido and arousal, erectile dysfunction, and ejaculatory or orgasmic disturbances; in some cases, endocrine-related effects such as gynecomastia have also been reported (13).

Earlier systematic reviews and meta-analyses yielded inconsistent estimates of sexual adverse events associated with the use of 5-ARIs, partly due to differences in study design, populations, dosing, and outcome definitions (14, 15). The most comprehensive meta-analysis to date, conducted by Lee et al. (16) and encompassing 15 placebo-controlled RCTs in male AGA patients (*n* = 4,495), reported a pooled relative risk of sexual dysfunction of 1.57 (95% CI 1.19–2.08) (16). However, that analysis was limited to studies published up to 2018 and did not include topical finasteride formulations, female patients with FPHL, or pharmacovigilance data on persistent sexual dysfunction following treatment discontinuation. Therefore, there is a need to re-analyze the available studies to accurately determine the incidence of adverse reactions. Quantifying these risks is clinically relevant for patient counseling, shared decision-making, and pharmacovigilance—particularly because AGA treatment is elective and long-term. In this context, we conducted a systematic review assessing the impact of 5-ARIs (finasteride and dutasteride) on the development of sexual dysfunction, emphasizing the importance of sexual health in the overall wellbeing of patients with AGA.

## 2 Material and methods

The search was performed in accordance with the PRISMA guidelines (17), ensuring a standardized and transparent approach to identifying, selecting, and evaluating studies relevant to the association between 5-ARIs and sexual dysfunction in AGA. The protocol defined the databases to be searched, the search terms to be used, the inclusion and exclusion criteria, and the procedures for study selection and data extraction. This systematic review was not prospectively registered in PROSPERO.

### 2.1 Search strategy

The information search was conducted in online databases containing large repositories of scientific research, namely MEDLINE, Scopus, Web of Science and Google Scholar, which were systematically searched in December 2025. The search was limited to articles published in English and up to December 2025. The study also excluded articles for which full access could not be obtained.

The search strategy was developed based on the research objective and included the following keywords: (“5α-reductase inhibitors” or “5-alpha reductase inhibitors” or “5-ARIs” or “finasteride” or “dutasteride”) AND (“side effects” or “sexual side effects” or “sexual adverse effects” or “sexual” or “sexual dysfunction” or “erectile dysfunction” or “decreased libido” or “loss of libido” or “ejaculatory disorders” or “post-finasteride syndrome” or “persistent sexual dysfunction”) AND (“androgenetic alopecia” or “male pattern hair loss” or “female pattern hair loss”).

Two independent reviewers (AZ and PKK) performed a comprehensive and unbiased literature search. In addition, the reference lists of relevant articles were manually screened, and a thorough search of related literature was conducted to ensure completeness. The search process was repeated prior to the final analysis to capture any newly published studies.

### 2.2 Data collection and eligibility criteria

The data collection process was conducted manually through content analysis.

The inclusion criteria (IC) that guide the preparation of this systematic literature review are explained as follows:

IC1: All original, peer-reviewed studies published in English.

IC2: Studies investigating sexual side effects in patients with AGA using 5-ARIs.

IC3: Studies employing quantitative, qualitative, or mixed-methods research designs.

The exclusion criteria (EC) were defined as follows:

EC1: Ineligible study design, including single case reports (*n* < 3), literature or systematic reviews, animal studies, and unpublished data.

EC2: Ineligible interventions and outcome measures, such as studies assessing only dermatological outcomes of androgenetic alopecia without evaluating sexual adverse events, studies reporting sexual dysfunction outcomes without concurrent assessment of drug exposure or treatment duration, and studies lacking appropriate statistical analysis or quantitative reporting of sexual adverse events.

EC3: Ineligible study populations, including studies focused solely on children, or patients with hair loss due to causes other than androgenetic alopecia (e.g., chemotherapy-induced alopecia, alopecia areata, trichotillomania), or patients with pre-existing sexual dysfunction unrelated to treatment.

The PICOS acronym (Population, Intervention, Comparison, Outcome, and Study design) was used to systematically analyze the research question (Table 1). The population of interest included adults diagnosed with AGA who were treated with 5-alpha reductase inhibitors (e.g., finasteride or dutasteride). Studies reporting on adverse effects associated with this therapy were included. Mixed populations were considered eligible if data for AGA patients receiving 5-alpha reductase inhibitors could be extracted separately or constituted at least 80% of the study sample. The intervention group was not mandatory. Observational and interventional studies that present predefined outcomes were included in the analysis. Eligible comparators included placebo, active comparators (e.g., minoxidil, alternative 5-ARI dose or formulation), and no separate control group; single-arm studies were eligible provided that sexual adverse events were quantitatively reported in relation to drug exposure.

**TABLE 1:** Eligibility criteria agccording to the PICO framework.

PICO element	Inclusion criteria	Exclusion criteria
Population	Adult patients ( ≥ 18 years) diagnosed with androgenetic alopecia (AGA), including male pattern hair loss (MPHL) and female pattern hair loss (FPHL). Mixed populations eligible if AGA patients on 5-ARIs constituted ≥ 80% of the sample	Children; patients with hair loss due to causes other than AGA (e.g., alopecia areata, chemotherapy-induced alopecia, trichotillomania); patients with pre-existing sexual dysfunction unrelated to treatment
Intervention	Systemic or topical 5-alpha reductase inhibitors (finasteride or dutasteride) at any dose and duration, used for the treatment of AGA	5-ARIs used exclusively for benign prostatic hyperplasia or other non-AGA indications without extractable AGA subgroup data
Comparison	Any comparator, including placebo, active comparator, or no control group	–
Outcome	Any sexual adverse effect reported as an outcome, including but not limited to decreased libido, erectile dysfunction, ejaculatory disorders, orgasmic dysfunction, genital hypoesthesia, and persistent sexual dysfunction (post-finasteride syndrome)	Studies reporting only dermatological outcomes without any assessment of sexual adverse effects
Study design	Original peer-reviewed studies published in English: RCTs, observational studies (cohort, case-control, cross-sectional), open-label clinical studies, case series ( ≥ 5 patients), surveys, pharmacovigilance analyses. One systematic review with meta-analysis (18) was additionally included as a supplementary source given its pooled quantitative estimates, clearly distinguished from primary studies in the analysis	Single case reports; animal studies; unpublished data; conference abstracts; studies without full-text access

#### 2.2.1 Evidence tiering

Studies were classified into two evidence tiers. The primary evidence tier included studies that explicitly investigated patients with androgenetic alopecia (AGA), male pattern hair loss (MPHL), or female pattern hair loss (FPHL), in which sexual adverse events were reported for the AGA-specific population. The supporting/indirect evidence tier included studies that informed the broader mechanistic, pharmacokinetic, or comparative-safety context but did not exclusively investigate AGA populations—namely, pharmacokinetic studies in healthy male volunteers, trials in women with hirsutism or hyperandrogenism (without FPHL diagnosis), pharmacovigilance or registry analyses of mixed-indication 5-ARI users, and systematic reviews/meta-analyses that pooled BPH and AGA cohorts or focused on BPH alone. These supporting studies were retained because they provide essential context for interpreting AGA-specific findings (e.g., dose-response, scalp vs. serum DHT kinetics, background event rates in non-AGA populations), but they were analyzed and reported separately from the primary synthesis. The tier classification for each included study is presented in Table 5.

### 2.3 Data extraction

After removing duplicate records, titles and abstracts were screened by the first author (AZ) and subsequently assessed by the second author (PKK) to identify potentially eligible studies. Full texts of selected articles were then reviewed in detail, and studies were included or excluded based on predefined criteria. Only studies reporting sexual side effects in patients receiving 5-alpha-reductase inhibitors for androgenetic alopecia were considered. Extracted data included: author(s), publication year, study design, sample size and characteristics (age, gender), intervention details (type and duration of 5-alpha-reductase inhibitor therapy), outcome measures (type of sexual side effects assessed and methods of assessment), and main findings.

### 2.4 Quality assessment of included studies

The methodological quality and risk of bias of the included studies were assessed using appropriate, widely accepted tools adapted to the type of publications analyzed. Observational studies were assessed using the Newcastle–Ottawa Scale (NOS), which encompasses domains related to participant selection, group comparability, and outcome assessment. For randomized controlled trials, the Risk of Bias 2 (RoB 2) tool was used to assess the risk of bias in key areas such as the randomization process, deviations from intended interventions, missing data, outcome measurement and selective reporting of results. The quality of systematic reviews and meta-analyses was assessed using AMSTAR 2, a comprehensive tool for assessing the methodological validity of these publications.

The assessment was conducted using dedicated, structured analytical tools to support the standardization of the assessment process and minimize the risk of interpretation errors. The results of the quality and risk of bias assessments were summarized in tabular form and considered in the interpretation of the obtained results.

Potential sources of bias in the identified studies are acknowledged including the small size of patient cohorts and heterogeneous populations. Efforts were made to minimize it through a comprehensive search strategy, predefined inclusion criteria, and critical appraisal of the included studies.

**FIGURE 1:**
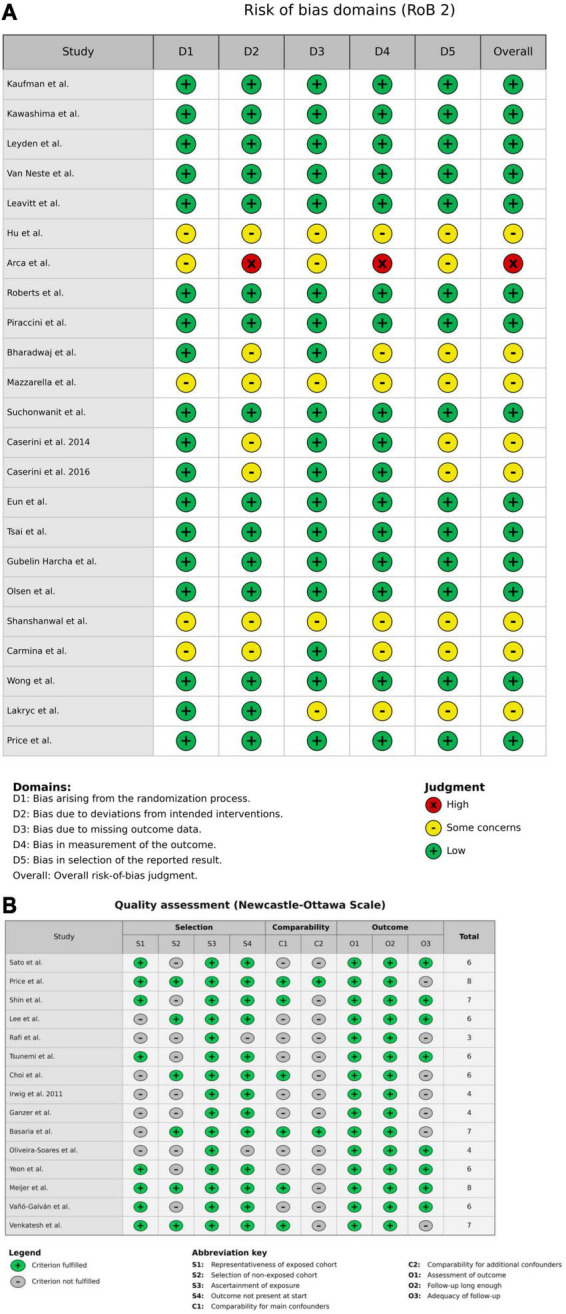
Risk of bias across randomized **(A)** and observational studies **(B)**.

The quality and risk of bias assessments of the included studies are summarized in Figure 1 and Tables 2–4.

**TABLE 2:** Quality assessment of randomized controlled trials (RoB 2).

Quality assessment of randomized controlled trials (RoB 2)					
	Author of the study	Year	Study type	Quality score	Key methodological note
1	K. D. Kaufman et al	1998	RCT (DB, PC)	1	High quality; seminal RCT.
2	M. Kawashima et al	2004	RCT (DB, PC)	1	Low risk; well-blinded.
3	J. Leyden et al	1999	RCT (DB, PC)	1	High quality; focused on frontal area.
4	D. Van Neste et al	2000	RCT (DB, PC)	1	High quality; objective metrics used.
5	M. Leavitt et al	2005	RCT (DB, PC)	1	Low risk; specific surgical context.
6	R. Hu et al	2015	RCT (Comparative)	2	Some concerns (Open-label).
7	E. Arca et al	2004	RCT (Comparative)	3	High risk (Lack of blinding/placebo).
8	J. L. Roberts et al	1999	RCT (Dose-ranging)	1	Low risk; clear dose-response data.
9	B. M. Piraccini et al	2022	RCT (Phase III)	1	High quality; modern Phase III trial.
10	A. V. Bharadwaj et al	2023	RCT (Open-label)	2	Some concerns (Small pilot study).
11	G. F. Mazzarella et al	1997	RCT (PC)	2	Some concerns (Early reporting standards).
12	P. Suchonwanit et al	2018	RCT (DB, PC)	1	Low risk; strong internal validity.
13	M. Caserini et al	2014	RCT (PK/PD)	2	Some concerns (Small sample/PK focus).
14	M. Caserini et al	2016	RCT (PK/PD)	2	Some concerns (Focused on DHT levels).
15	H. C. Eun et al	2010	RCT (Phase III)	1	High quality; Phase III Dutasteride trial.
16	T. F. Tsai et al	2018	RCT (Prospective)	1	Low risk; robust sexual function data.
17	W. Gubelin Harcha et al	2014	RCT (Phase III)	1	High quality; precise dose comparison.
18	E. A. Olsen et al	2006	RCT (Comparative)	1	Low risk; direct Fin vs. Dut comparison.
19	S. J. Shanshanwal et al	2017	RCT (Evaluator-blind)	2	Some concerns (Evaluator-blinded only).
20	E. Carmina et al	2003	RCT (Comparative)	2	Some concerns (Small female cohort).
21	I. L. Wong et al	1995	RCT (Comparative)	1	High quality; well-controlled (hirsutism).
22	E. M. Lakryc et al	2003	RCT (Open-label)	2	Some concerns (Open-label nature).
23	V. H. Price et al	2000	RCT (DB, PC)	1	High quality; definitive negative result.

Risk levels: Low (1), Some Concerns (2), High (3). For this table, a lower score indicates higher quality.

**TABLE 3:** Systematic reviews and meta-analyses (AMSTAR 2).

Systematic reviews and meta-analyses (AMSTAR 2)				
	Author of the study	Year	Quality score	Key review findings
1	J. Wang et al	2018	8	High quality; systematic meta-analysis of 5-ARI risks.

Score: High Quality (9), Moderate (6), Low (3).

**TABLE 4:** Quality assessment of observational, cohort studies and survey studies (NOS).

Quality assessment of observational, cohort studies and survey studies (NOS).					
	Author of the study	Year	Study design	Quality score	Evidence level
1	A. Sato et al	2012	Long-term observ.	6	Moderate
2	V. H. Price et al	2006	Extension study	8	High
3	J. W. Shin et al	2019	Retrospective	7	High
4	S. G. Lee et al	2019	Retrospective	6	Moderate
5	A. W. Rafi et al	2011	Case series	3	Low
6	Y. Tsunemi et al	2016	Open-label Obs.	6	Moderate
7	G. S. Choi et al	2022	Chart review	6	Moderate
8	M. S. Irwig et al	2011	Cross-sectional	4	Moderate/Low
9	C. A. Ganzer et al	2015	Survey	4	Moderate/Low
10	S. Basaria et al	2016	Case-control	7	High
11	R. Oliveira-Soares et al	2013	Case series	4	Low
12	J. H. Yeon et al	2011	Open-label clinical trial	6	Moderate
13	M. Meijer et al	2018	Population-based cohort study	8	High
14	S. Vañó-Galván et al	2020	Retrospective	6	Moderate
15	T. Venkatesh et al	2025	Retrospective	7	High

Scoring: 7–9 points (High Quality), 4–6 points (Moderate), < 4 points (Low).

### 2.5 Data synthesis

**FIGURE 2:**
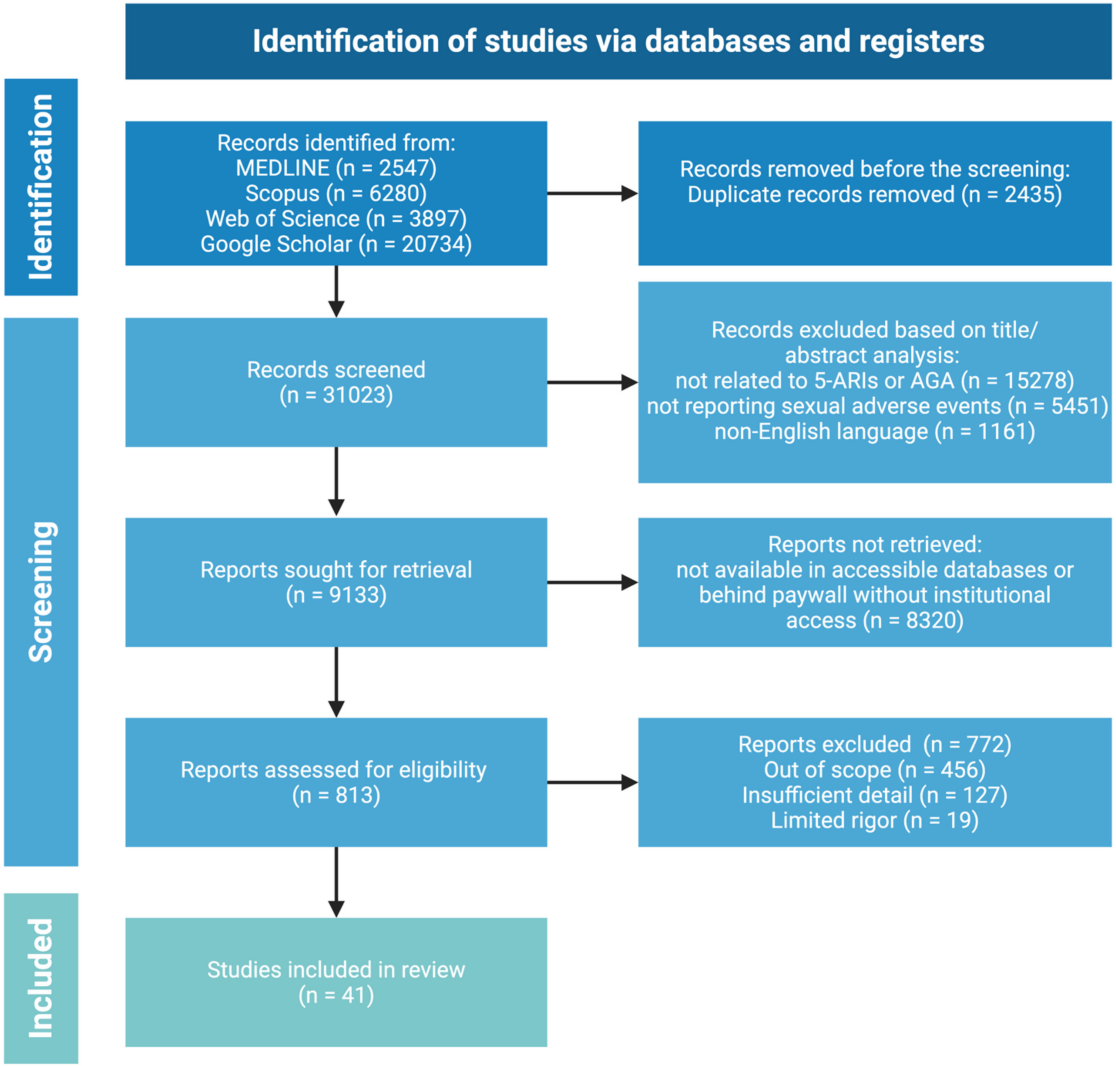
Literature search carried out according to the PRISMA guidelines.

In total, 41 articles were included, classified into 33 primary-evidence studies and 8 supporting-evidence studies. A PRISMA diagram was generated to visually represent the entire search strategy and the subsequent screening and inclusion process. The search strategy is presented in Figure 2. Ethics approval was not required for this study.

## 3 Results

Initial database searches (MEDLINE, Scopus, Web of Science, Google Scholar) identified a total of 33 458 records. After removing 2,435 duplicates, 31,023 records qualified for the initial screening stage. Based on title and abstract analysis, 21,890 records were excluded, leaving 9,133 reports requiring full review. Of these, full text could not be retrieved for 8,320 records. Final eligibility assessment was performed on 813 full-text articles. After excluding 772 reports that did not meet the criteria, 41 studies were included in the final systematic review, comprising 33 primary-evidence studies in AGA, MPHL, or FPHL populations and 8 supporting-evidence studies providing mechanistic, pharmacokinetic, or comparative-safety context (Figure 2).

Table 5 provides a summary of all 41 studies included in the review, classified according to the following parameters: study design (e.g., RCT, cohort study), population, participant demographic characteristics with particular emphasis on mean age, type of 5-alpha-reductase inhibitor used (finasteride vs. dutasteride), dosing regimen, and key outcomes related to sexual dysfunction.

**TABLE 5:** Summary of the results of all studies included in the systematic review.

	Author of the study	Study type	Population	Age	Evidence tier	Medication	Dose	Study duration	Sexual side effects	Sexual side effects placebo group
1	K. D. Kaufman et al. (19, 20)	Double-blind, placebo-controlled, randomized clinical trial	1,553 Men with AGA	18–41 years	Primary	Finasteride (oral)	1 mg daily	2 years (24 months)	After 12 months: decreased libido 1.9%, erectile dysfunction 1.4%, ejaculation disorder 1%. Year 2 (new users): erectile dysfunction 1.1%, decreased libido 1.3%.	After 12 months: decreased libido 0.9%, erectile dysfunction 1.3% ejaculation disorder 0.4 %.
2	A. Sato et al. (21)	Retrospective, open-label, multi-center study	3,177 Japanese men with AGA	37.5 ± 11.9 years	Primary	Finasteride (oral)	1 mg daily	Up to 42 months (3.5 years)	Decreased libido was reported in only 8 patients (0.25%)	N/A–No control group
3	M. Kawashima et al. (22)	Double-blind, randomized, placebo-controlled study	414 Japanese men with AGA	18–40 years	Primary	Finasteride (oral)	1 mg vs. 0.2 mg	1 year (48 weeks)	Finasteride 1 mg: Decreased libido (2.9%) Finasteride 0.2 mg: Decreased libido (1.5%)	Decreased libido (2.2%)
4	J. Leyden et al. (23)	Double-blind, randomized, placebo-controlled study	326 Men with frontal male pattern hair loss	18–40 years	Primary	Finasteride (oral)	1 mg daily	1 Year (12 months)	Sexual adverse events (2%) in the finasteride group. 2 patients reported decreased libido and 1 patient reported impotence.	Sexual adverse effects (2%). 2 patients reported decreased libido and 1 patient reported an ejaculation disorder.
5	D. Van Neste et al. (24)	Randomized, double-blind, placebo-controlled study	212 Men with AGA	18–40 years	Primary	Finasteride (oral)	1 mg daily	48 weeks	Sexual adverse events (1.9%).	Sexual adverse effects (0.9%)
6	V. H. Price et al. (25)	Double-blind, randomized, placebo-controlled study	166 Men with AGA	22–40 years	Primary	Finasteride (oral)	1 mg daily	4 years	Decreased libido was reported by only one patient in the finasteride group during the initial 48-week study period and again during the second study extension.	Not reported.
7	M. Leavitt et al. (26)	Double-blind, randomized, placebo-controlled, multi-center study	79 Men undergoing hair transplantation	20–45 years	Primary	Finasteride (oral)	1 mg daily	14 months (started 4 weeks before surgery)	Decreased libido 2.5% Erectile dysfunction 2.5%	Not reported.
8	R. Hu et al. (27)	Randomized, comparative clinical study	450 Chinese men with AGA	18–50 years	Primary	Finasteride (oral) Minoxidil (topical)	1 mg daily (Finasteride) + 5% Minoxidil twice daily	1 year (12 months)	Sexual side effects occurred in 1.8% of the finasteride group: decreased libido (0.9%), erectile dysfunction (0.3%), testicular pain (0.3%). All cases were mild and reversible.	N/A–No control group
9	E. Arca et al. (28)	Open, randomized, comparative study	65 Men with AGA	18–45 years	Primary	Finasteride (oral) vs. 5% Topical Minoxidil	1 mg daily (Finasteride group)	1 year (12 months)	Decreased libido was noted in 6 patients in the finasteride group.	N/A–No control group
10	J. W Shin et al. (29)	Retrospective study	126 Men with AGA	18–40 years	Primary	Finasteride (oral)	1 mg daily	5 years	Over a 5-year period, 10 patients (7.9%) reported sexual dysfunction: decreased libido 3.1%, erectile dysfunction 2.4%, decreased semen volume or watery semen 2.4%,	N/A–No control group
11	J. L. Roberts et al. (30)	Multicenter, 1-year, randomized placebo-controlled study	*n* = 575 Men aged 18–36 years with vertex MPHL (Hamilton-Norwood IIIv–V); two parallel RCTs (*n* = 249 dose-ranging; *n* = 326 dose-confirmation).	18–36 years	Primary	Finasteride (oral)	0.01 mg, 0.2 mg, 1 mg, and 5 mg daily	1 year (12 months)	Finasteride (5 mg): Sexual adverse effects (3.6%) Decreased libido (2.7%) Erectile dysfunction (1.8%) Finasteride (1 mg): Sexual adverse effects (4.3%) Decreased libido (1.7%) Erectile dysfunction (2.6%)	Sexual adverse effects (3.4%) Decreased libido (3%) Erectile dysfunction (0%)
12	B. M. Piraccini et al. (32)	Randomized, double-blind, placebo-controlled, phase III trial	458 Men with AGA	Mean age: 32 years	Primary	Topical finasteride (0.25%) vs. Oral Finasteride	1–4 Sprays (50–200 μL) daily or 1 mg daily	6 months (24 weeks)	Sexual adverse events (sexual dysfunction, erectile dysfunction, decreased libido, loss of libido) was observed in 2.8% in the topical finasteride group and 4.8% in oral finasteride.	Sexual adverse events (sexual dysfunction, erectile dysfunction, decreased libido, loss of libido) was observed in 3.3% placebo.
13	A. V. Bharadwaj et al. (33)	Single center, pilot, randomized, open-label study	60 Men with AGA	18–40 Years	Primary	Topical Finasteride (0.25%) + Minoxidil (5%)	1 mL twice daily	6 months (24 weeks)	No instances of sexual dysfunction reported.	N/A–No control group
14	G. F. Mazzarella et al. (34)	Single-blind, placebo-controlled study	52 Patients with AGA	18–38 Years	Primary	Topical finasteride (0.005% solution)	1 mL twice daily	16 months	No instances of sexual dysfunction reported.	No instances of sexual dysfunction reported.
15	P. Suchonwanit et al. (35)	Randomized, double-blind, controlled study	40 Men with AGA	18–60 Years	Primary	Topical finasteride (0.25%) + Minoxidil (3%)	1 mL twice daily	6 months (24 weeks)	No instances of sexual dysfunction reported.	N/A–No control group
16	M. Caserini et al. (36)	Randomized, single center, open-label, parallel-group, exploratory study,	24 Healthy male volunteers	18–65 Years	Supporting	Topical finasteride (0.25% solution) vs. Oral Finasteride	2.275 mg b.i.d. (14 doses total) or 1 mg o.d. (7 doses total)	7 days	There were no changes in testosterone levels in any patient in either group. In addition, none of the patients reported sexual side effects.	N/A–No control group
17	M. Caserini et al. (37)	Randomized, parallel-group, 1-week study	50 Healthy men (Study I: *n* = 18; Study II: *n* = 32)	18–65 Years	Supporting	Topical finasteride (0.25%) vs. Oral Finasteride	100 μL, 200 μL, 300 μL, 400 μL, 1 mL (topical) or 1 mg (oral)	1 week	No significant changes in serum testosterone levels were observed. It was noted that 0.25% finasteride applied once daily at doses of 100 and 200 μL is effective in inhibiting DHT.	N/A–No control group
18	A. W. Rafi et al. (38)	Open-label pilot study	15 Patients with AGA and Atopic conditions	24–72 Years	Primary	Combination (Topical Finasteride, Minoxidil, Ketoconazole, etc.)	Compounded topical formula	9 months	No sexual side effects were reported.	N/A–No control group
19	H. C. Eun et al. (39)	Randomized, double-blind, placebo-controlled, phase III study	153 Men with AGA	18–49 years	Primary	Dutasteride (oral)	0.5 mg daily	6 months (26 weeks)	Sexual adverse events (4.1%) sexual dysfunction (4.1%) Erectile dysfunction (0%) Ejaculation disorder (0%)	Sexual adverse events (4%) sexual dysfunction (2.7%) Erectile dysfunction (1.3%) Ejaculation disorder (1.3%)
20	Y. Tsunemi et al. (40)	Open-label, long-term, multi-center study	120 Men with AGA	20–50 years	Primary	Dutasteride (oral)	0.5 mg daily	1 year (52 weeks)	Erectile dysfunction (11.7%) Decreased libido (8.3%), Ejaculatory dysfunction (4.2%), Sexual dysfunction (11.7%)	N/A–No control group
21	T. F. Tsai et al. (41)	Prospective, randomized, multi-center study	117 Men with AGA	23–50 Years	Primary	Dutasteride (oral)	0.5 mg daily	6 months (24 weeks)	Erectile dysfunction (12%) Decreased libido (2%), Ejaculatory dysfunction (2%),	Erectile dysfunction (5%) Decreased libido (3%), Ejaculatory dysfunction (0%),
22	W. Gubelin Harcha et al. (42)	Randomized, double-blind, active- and placebo-controlled study	917 Men with AGA	20–50 Years	Primary	Dutasteride (oral) vs. Finasteride (oral) vs. Placebo	Dutasteride (0.02, 0.1, 0.5 mg) vs. Finasteride (1 mg)	6 months (24 weeks)	Finasteride (1 mg): Altered libido (6.7%) Impotence (6.1%) Ejaculation disorders (3.9%) Dutasteride (0.5 mg): Altered libido (4.9%) Impotence (5.4%) Ejaculation disorders (3.3%)	Altered libido (1.7%) Impotence (3.9%) Ejaculation disorders (3.3%)
23	E. A. Olsen et al. (43)	Randomized, placebo-controlled study	416 Men with AGA	21–45 Years	Primary	Dutasteride (oral) vs. Finasteride (oral) vs. Placebo	Dutasteride (0.05, 0.1, 0.5, 2.5 mg) vs. Finasteride(5 mg)	6 months (24 weeks)	Finasteride (5 mg): Decreased libido (4%) Ejaculation disorders (3%) Impotence (1%) Dutasteride (2.5 mg): Decreased libido (13%) Ejaculation disorders (1%) Impotence (0%) Dutasteride (0.5 mg): Decreased libido (1%) Ejaculation disorders (1%) Impotence (0%)	Decreased libido (3%) Ejaculation disorders Impotence (5%)
24	G. S. Choi et al. (44)	Retrospective, multicentre chart review study	600 Men with AGA	Dutasteride: 41.7 ± 10.6 Finasteride: 36. 8 ± 11.2	Primary	Dutasteride (oral) vs. Finasteride (oral)	Dutasteride 0.5 mg vs. Finasteride 1 mg	Mean 3 years (up to 5 years)	Finasteride (5 mg): Sexual adverse events (1.1%) Decreased libido (0.7%) Impotence (0%) Other sexual dysfunction (0.4%) Dutasteride (2.5 mg): Sexual adverse events (1.6%) Decreased libido (1.2%) Impotence (0.4%) Other sexual dysfunction (0%)	N/A–No control group
25	S. J. Shanshanwal et al. (45)	Randomized, open-label, evaluator-blinded study	90 Men with AGA	18–40 Years	Primary	Dutasteride (oral) vs. Finasteride (oral)	Dutasteride 0.5 mg vs. Finasteride 1 mg	6 months (24 weeks)	Finasteride (5 mg): Erectile dysfunction (0.11%) Decreased libido (0.33%) Dutasteride (0.5 mg): Erectile dysfunction (0.33%) Decreased libido (0.44%)	N/A–No control group
26	S. G. Lee et al. (48)	Retrospective clinical study	1,673 (total) Finasteride (*n* = 52): continued pre- and post-mastectomy	25–30 years	Primary	Finasteride (oral)	1 mg daily	The median duration of finasteride therapy before and after surgery was 12 (5–25.75) and 33 (27.5–40.5) months, respectively.	No patients taking finasteride after mastectomy developed recurrence. These findings indicate that finasteride used for alopecia does not influence recurrence rates following complete mastectomy.	None of the patients who did not take finasteride had a relapse.
27	M. S. Irwig et al. (50)	Observational study (interviews)	71 Healthy men reporting persistent side effects	21–46 years	Primary	Finasteride (oral)	1 mg, 5 mg		Persistent sexual dysfunction was reported by subjects using finasteride, characterized by high rates of low libido (94%), erectile dysfunction (92%), decreased arousal (92%), and orgasmic dysfunction (69%).	N/A–No control group
28	C. A. Ganzer et al. (51)	Online survey	131 Men reporting persistent symptoms	21–62 years	Primary	Finasteride (oral)	1 mg		Decreased sex drive (93%) Complete impotence (40%) Intermittent erectile dysfunction (83%) Depressed affect (73%) Suicidal ideations (63%)	N/A–No control group
29	S. Giatti et al. (52)	Online survey	54 Men reporting persistent symptoms	23–55 years	Primary	Finasteride (oral)	1 mg, 1.25 mg		It was reported higher post-treatment reporting rates compared with on- treatment reporting for several sexual symptoms, including perceived loss of “brain–penis connection” (59% vs. 22%), loss of libido (56% vs. 24%), erectile difficulties (61% vs. 17%), and genital numbness/paresthesia (37% vs. 17%)	N/A–No control group
30	S. Basaria et al. (53)	Retrospective, placebo-controlled study	56 Men reporting persistent symptoms	18–50 years	Primary	Finasteride (oral)	1 mg		Men with PFS reported significantly lower sexual function scores (IIEF), but the study found no differences in testosterone levels or androgen receptor density between the groups.	Sexual desire (29.5 on MSHQ)
31	T. Kiguradze et al. (54)	Stratified, multivariable quasi-experimental cohort study	11,909 men with 5 ARIs exposure	No specific age restriction	Supporting	Finasteride (oral) Dutasteride (oral)	No specific dose restriction		Among men exposed to 5α-RIs, 1.4% (167/11,909) developed persistent erectile dysfunction (PED); median persistence 1,348 days after discontinuation (IQR 632–2,321). Of men with new-onset ED, 31.5% (167/530) progressed to PED. Combined NSAID use and > 208.5 days of 5α-RI exposure was associated with a 4.8-fold increased risk of PED (NNH 59.8; *p* < 0.002). Among men aged 16–42 years exposed to finasteride ≤ 1.25 mg/day, 0.8% (34/4,284) developed PED; median persistence 1,534 days (IQR 651–2,351). In this younger group, 33% (34/103) of men with new-onset ED developed PED. Finasteride exposure > 205 days in young men was associated with a 4.9-fold increased risk of PED (NNH 108.2; *p* < 0.004).	N/A–No control group
32	R. Oliveira-Soares et al. (58)	Prospective, open-label study	40 Postmenopausal women with FPHL	Post- menopausal	Primary	Finasteride (oral)	5 mg daily	18 months	No sexual side effects were reported.	N/A–No control group
33	J. H. Yeon et al. (59)	Randomized, open-label study	87 Normoandrogenic Asian women with FPHL	21–69 years	Primary	Finasteride (oral)	5 mg daily	12 months	No sexual side effects were reported.	N/A–No control group
34	E. Carmina et al. (60)	Comparative clinical study	48 Hyperandrogenic women	25 ± 2 Years	Supporting	Finasteride (oral) vs. Flutamide vs. Cyproterone acetate	5 mg daily (Finasteride)	12 months	No significant side effects were reported in the finasteride group.	N/A–No control group
35	I. L. Wong et al. (62)	Prospective randomized trial	40 Hirsute women	15–40 years	Supporting	Finasteride (oral) vs. Spironolactone	5 mg daily (Finasteride)	6 months	No sexual side effects were reported in the finasteride group.	N/A–No control group
36	E. M. Lakryc et al. (61)	Randomized, double-blind, placebo-controlled study	24 Hirsute women	19–40 years	Supporting	Finasteride (oral)	5 mg daily	6 months	No sexual side effects were reported.	No sexual side effects were reported.
37	V. H. Price et al. (63)	Randomized, double-blind, placebo-controlled study	137 Postmenopausal women with FPHL	41–60 years	Supporting	Finasteride (oral)	1 mg daily	12 months	No sexual side effects were reported.	No sexual side effects were reported.
38	M. Meijer et al. (64)	Register based cohort study	No specific age restriction	N/A (General male population using finasteride)	Supporting	Finasteride (oral)	1 mg and 5 mg	Prescription records from 1995 to 2010 (varying by country), with follow-up up to 15 years	Not evaluated.	Not evaluated.
39	J. Wang et al. (18)	Systematic review and meta-analysis	Data from multiple observational studies	Mean age range from 60 to 73.2 years old	Supporting	Finasteride (oral) and Dutasteride (oral)	5 mg	Meta-analysis of long-term data	Not evaluated.	Not evaluated.
40	S. Vañó-Galván et al. (65)	Retrospective, monocentric, descriptive study	307 Men with AGA	18–79	Primary	Dutasteride (oral)	0.5 mg daily	12 months	Dutasteride-related adverse effects (AE) were observed in 20 out of 307 patients (6.5%): decreased libido (*n* = 9), erectile dysfunction (*n* = 4), mood disorders (*n* = 3), gynecomastia (*n* = 2) and lower ejaculation volume (*n* = 2).	Not evaluated.
41	T. Venkatesh et al. (66)	Retrospective cohort study	810 Women	47–70	Primary	Finasteride (oral) or Dutasteride (oral)	0,5 mg, 1 mg, 5 mg daily	Median duration of follow-up after first prescription was 3.7 years	Not evaluated.	Not evaluated.

### 3.1 Finasteride 1 mg

Across placebo-controlled studies of finasteride 1 mg/day in men with AGA, sexual adverse events were reported at low frequencies and typically included decreased libido, erectile dysfunction, and reduced ejaculate volume. In two 12-month randomized, double-blind trials with a blinded extension, Kaufman et al. (19, 20) reported at month 12 erectile dysfunction, decreased libido, and reduced ejaculate volume in 1.4, 1.9, and 1.0% of finasteride-treated participants, respectively, compared with 0.9, 1.3, and 0.4% in the placebo group. During the extension phase, sexually related adverse events were mainly reported among participants who initiated finasteride only in year 2; no such events were reported among those who discontinued finasteride (19). The findings indicate a comparable incidence of adverse effects in both groups, with symptoms occurring mainly in the context of prolonged exposure, suggesting a possible association with treatment duration.

In an extensive Japanese post-marketing/clinical study, Sato et al. (21) reported adverse events in 0.7% of participants overall; decreased libido occurred in 0.25% (8/3,177), and three of these participants discontinued treatment based on risk–benefit considerations (21).

Consistent findings were reported in other randomized, placebo-controlled studies. In a 12-month multicenter trial with an extension year, Kawashima et al. (22) found no meaningful difference between finasteride and placebo in the overall frequency of sexual adverse events (2% in each group); events included decreased libido, ejaculation disorder, and impotence, and no participant discontinued because of these symptoms (22). In a separate 1-year placebo-controlled study in men with frontal scalp thinning, Leyden et al. (23) likewise reported similar rates of sexual adverse events in the finasteride and placebo groups (2.0% vs. 2.0%); events were reported to resolve during continued therapy or after discontinuation, although the authors noted limitations related to sample size and event frequency estimates (23). Despite the low and comparable incidence of symptoms in both groups and their resolution, the small sample size warrants cautious interpretation of the results.

In a 48-week multicenter RCT, Van Neste et al. (24) reported sexual dysfunction in 1.9% of finasteride-treated participants and 0.9% of placebo-treated participants; none discontinued, and symptoms were reported to resolve during or shortly after treatment. In a long-term single-center placebo-controlled study over 4 years, Price et al. (25) reported high tolerability, with decreased libido reported by one participant in the finasteride group during the initial period and again during the extension.

In the peri-transplant setting (randomized, placebo-controlled, Leavitt et al. (26) reported reduced libido and erectile dysfunction in the finasteride group, with no discontinuations attributed to these events. In a 12-month randomized comparative study, assessing finasteride, topical minoxidil, or combination therapy, Hu et al. (27) reported sexually related adverse events in a small proportion of finasteride-exposed participants (including combination therapy), most commonly decreased libido; these events were described as mild and resolving after discontinuation. The findings indicate that adverse effects occurred relatively infrequently in the studied population.

In an open-label randomized study comparing finasteride 1 mg/day with topical minoxidil, Arca et al. (28) reported decreased libido in several finasteride-treated participants, with resolution after treatment; the authors also noted changes in serum androgen levels (increased total testosterone and reduced free testosterone). Finally, in a retrospective cohort with 5-year follow-up, Shin et al. (29) reported sexual dysfunction in 7.9% of participants, most commonly decreased libido, erectile dysfunction, and reduced/altered semen volume; two participants temporarily interrupted therapy. The findings indicate that the observed symptoms were transient, suggesting their clinical reversibility.

### 3.2 Finasteride 5 mg versus 1 mg

Roberts et al. (30) evaluate the efficacy and safety of finasteride in men aged 18–36 years with androgenetic alopecia using two randomized, double-blind, placebo-controlled trials. In the dose-ranging study, drug-related sexual adverse events were reported in 4.3% (1 mg), 6.1% (0.2 mg), and 1.7% (0.01 mg) of participants, with decreased libido reported in 1.7% (1 mg), 3.5% (0.2 mg), and 1.7% (0.01 mg), and erectile dysfunction in 2.6% (1 mg), 1.7% (0.2 mg), and 0% (0.01 mg). Overall, the authors reported that the incidence of sexually related adverse events was generally comparable to placebo and did not show a dose-dependent pattern or an increase with longer treatment duration up to 12 months (30).

### 3.3 Topical finasteride

Evidence comparing topical finasteride with oral finasteride suggests that topical formulations may achieve clinical benefit with lower systemic exposure (31). In a randomized, multicenter, double-blind, double-dummy, parallel-group trial, sexual adverse events were reported in 2.8% of participants receiving topical finasteride, 3.3% receiving placebo, and 4.8% receiving oral finasteride (32). Discontinuation due to sexual dysfunction occurred in 1.1% of placebo-treated patients and 2.4% of those receiving oral finasteride. Importantly, Sexual Dysfunction Questionnaire scores at weeks 12 and 24 did not differ significantly between groups (32). Pharmacokinetic findings supported substantially reduced systemic exposure with topical therapy (maximum serum concentration > 100-fold lower than with oral therapy), accompanied by a more minor mean reduction in serum DHT (34.5% vs. 55.6%). On this basis, the authors concluded that systemic sexual adverse effects related to DHT suppression may be less likely with topical than with oral finasteride (32).

Smaller comparative studies have generally reported few or no sexually related adverse events with topical finasteride regimens. In a pilot randomized open-label trial, no sexual adverse effects were reported in men treated with topical 5% minoxidil plus 0.25% finasteride, compared with minoxidil alone or finasteride alone (33). Similarly, a single-blind placebo-controlled 16-month trial of topical finasteride 0.005% found no changes in plasma total testosterone, free testosterone, or DHT, and no reported sexual side effects (34). The absence of significant changes in androgen levels suggests that finasteride does not substantially affect the hormonal profile in the studied population.

Combination topical therapy has also been evaluated. In a randomized, double-blind, controlled study of male patients, a topical solution containing 0.25% finasteride and 3% minoxidil was compared with 3% minoxidil alone. Median percentage reductions in plasma DHT at week 24 were minor and did not differ significantly between groups (5.7% vs. 7.4%; *p* = 0.92), and no sexual dysfunction was reported in either arm (35). These results are in agreement with previous studies in this field (35).

Pharmacodynamic and pharmacokinetic studies provide a mechanistic context for these observations. In an exploratory open-label parallel-group study, both topical finasteride 0.25% solution and oral finasteride 1 mg produced substantial short-term suppression of plasma DHT after 1 week (topical ∼68–75% vs. oral ∼62–72%), with no reported changes in testosterone levels and no sexual adverse events. Despite similar DHT suppression, topical drug concentrations remained significantly lower (*p* < 0.001) (36). Similar DHT suppression alongside lower drug concentrations with topical therapy suggests a potentially more favorable safety profile (36, 37).

Dose-ranging studies suggest that lower topical volumes of finasteride 0.25% solution can achieve meaningful scalp DHT suppression while limiting systemic exposure. In two randomized parallel-group trials, once-daily 1 mL topical finasteride reduced scalp DHT by 70%, whereas twice-daily application and oral finasteride 1 mg achieved reductions of 50%, with serum DHT decreasing by 60–70% across groups. In the dose-escalation arm (100–400 μL once daily), scalp DHT inhibition ranged from 37 to 54%, with the lowest systemic impact observed at 100–200 μL. Serum testosterone remained stable. These findings suggest that 100–200 μL once daily may provide sufficient scalp DHT inhibition with reduced systemic effects (37).

Finally, a small pilot study of a combined topical regimen (finasteride, dutasteride, and minoxidil in a hypoallergenic lotion) in men with AGA and atopy (*n* = 15; 9 months) reported clinical improvement without reported erectile dysfunction, fatigue, breast enlargement, or decreased semen volume (38). These observations are consistent with the hypothesis that topical treatment is associated with a lower risk of systemic effects.

### 3.4 Dutasteride 0.5 mg

An analysis of clinical trial results using dutasteride at a dose of 0.5 mg/day in men with androgenetic alopecia (AGA) indicates a variable incidence of sexual adverse events, while maintaining a high drug tolerability profile. In the 6-month, randomized, double-blind, phase III trial by Eun et al. (39) included men with AGA who received dutasteride 0.5 mg daily or placebo for 6 months, sexual dysfunction–related adverse events were the most commonly reported drug-related events, occurring in 4.1% of participants in the dutasteride arm and 2.7% in the placebo arm. Erectile dysfunction and an ejaculatory disorder were each reported by one participant in the placebo group. Overall, the between-group differences were not statistically significant (*p* > 0.05), and no participant discontinued treatment due to a sexual adverse event (39).

Longer-term safety data come from a multicenter, open-label, prospective outpatient study in Japan by Tsunemi et al. (40), in which 120 men received dutasteride 0.5 mg once daily for 52 weeks. Reported rates of erectile dysfunction, decreased libido, ejaculatory dysfunction, sexual dysfunction, and impotence were 11.7, 8.3, 4.2, 3.3, and 11.7%, respectively. Mean time to resolution in days was also reported. Notably, none of the participants withdrew from the study due to sexual adverse events (40).

In a study by Tsai et al. (41), 117 men with AGA were randomized to dutasteride 0.5 mg or placebo for a 24-week double-blind period, followed by a 24-week open-label extension in which all participants received dutasteride. During the double-blind phase, sexual adverse events were reported more frequently with dutasteride than placebo (16% vs. 8%). In the placebo group, erectile dysfunction, decreased libido, and ejaculatory dysfunction were reported in 5, 3, and 0% of participants, respectively, compared with 12, 2, and 2% in the dutasteride group. During the open-label period, the overall incidence of sexual adverse events decreased to 5%. Sexual symptoms resolved during treatment or after study completion, and no participant discontinued therapy because of adverse events (41). The study findings demonstrate full symptom resolution, supporting their reversible nature in the analyzed population.

### 3.5 Intralesional dutasteride

No studies reporting sexual adverse effects following intralesional dutasteride were identified in our search, reflecting the lack of available data for this administration route.

### 3.6 Finasteride versus dutasteride

A large, multicenter, randomized, double-blind, placebo-controlled trial compared dutasteride (0.02, 0.1, or 0.5 mg/day), finasteride (1 mg/day), and placebo in men with AGA (42). Overall, sexual adverse events were reported more frequently in the active-treatment arms (dutasteride and finasteride) than with placebo; however, no clear dose–response pattern across dutasteride doses was observed based on the reported event counts. Erectile dysfunction was reported in approximately 1% of participants in the placebo, dutasteride 0.1 mg, and finasteride groups, and decreased libido was reported in approximately 1% of participants in the placebo and dutasteride 0.1 mg groups (42). The authors did not provide separate symptom-specific percentages for the dutasteride 0.02 mg and 0.5 mg groups, but emphasized that symptom-level events were infrequent and did not differ meaningfully across doses.

Long-term, real-world safety has also been evaluated in a multicenter retrospective chart review from South Korea, including 600 adult men treated with dutasteride (*n* = 295) or finasteride (*n* = 305) for AGA (43). Adverse events were documented in 7.6% of patients receiving dutasteride and 10.5% of those receiving finasteride; the most commonly reported events were sexual in nature, including decreased libido and erectile dysfunction (43).

Additional comparative safety information comes from a randomized, multicenter trial in 416 men assigned to dutasteride (0.05, 0.1, 0.5, or 2.5 mg/day), finasteride 5 mg/day, or placebo for 24 weeks (44). Decreased libido was reported across all groups, with the highest number of cases observed in the dutasteride 2.5 mg arm; most cases resolved either during ongoing treatment or within several weeks after discontinuation (44). No apparent difference in impotence rates between groups was reported, and gynecomastia was rare (one case in the placebo group) (44). The findings indicate a comparable adverse event profile across groups, with a potential dose-related effect on decreased libido and a transient nature of most symptoms

Finally, an open-label, evaluator-blinded comparison in 900 men (18–40 years) treated for 24 weeks reported similarly low rates of erectile dysfunction and reduced libido in both the dutasteride 0.5 mg and finasteride 1 mg groups, with no statistically significant differences between treatments. (45) These observations are consistent with the majority of available studies.

### 3.7 The effect of finasteride on the development of gynecomastia

Gynecomastia affects body image and sexual attractiveness, which can lead to psychogenic sexual dysfunction. 5-alpha reductase inhibitors, by modifying the testosterone-to-estrogen ratio, may contribute to its development. Hormonal and endocrine effects, including gynecomastia, were less frequently reported (46, 47). Lee et al. (48) retrospectively assessed whether continuing finasteride after subcutaneous mastectomy with liposuction affects gynecomastia recurrence in men treated with finasteride for alopecia. Among 1,673 patients operated on between 2014 and 2016, 52 continued finasteride postoperatively. At 1-year ultrasonographic follow-up, postoperative complication rates and recurrence rates did not differ significantly between finasteride users and non-users, and no recurrences were observed in the finasteride group (48). The absence of gynecomastia recurrence in patients using finasteride suggests that the therapy is not associated with a significant risk of recurrence after surgical treatment.

### 3.8 Post-finasteride syndrome

The prevalence of persistent symptoms after finasteride exposure remains unknown, and available estimates are uncertain (49). In a study based on standardized interviews, Irwig and Kolukula (50) evaluated 71 men who reported persistent sexual symptoms for at least 3 months after discontinuing finasteride. Participants most frequently described reduced libido, erectile dysfunction, decreased arousal, and difficulty achieving orgasm. The mean duration of finasteride exposure was 28 months, and the mean duration of persistent sexual symptoms was 40 months at the time of interview (50). The observed duration indicates a potentially chronic course in selected cases.

Survey-based data have reported similar patterns but are inherently limited by selection and recall bias. In one pilot analysis of 131 online surveys from men reporting symptoms persisting at least 3 months after finasteride discontinuation, 84% indicated that symptoms were absent during treatment and appeared after stopping the drug (51). Frequently reported sexual complaints included decreased sex drive (93%), reduced semen volume/potency (82%), and gynecomastia (70%) (51). In another survey study, Giatti et al. (52) reported higher post-treatment reporting rates compared with on-treatment reporting for several sexual symptoms, including perceived loss of “brain–penis connection” (59% vs. 22%), loss of libido (56% vs. 24%), erectile difficulties (61% vs. 17%), and genital numbness/paresthesia (37% vs. 17%) (52). The increase in symptom frequency after treatment may suggest a complex relationship with therapy.

Despite these reports, the underlying pathophysiology of persistent symptoms remains incompletely characterized, and evidence regarding hormonal status, body composition, cognition, and mood is limited. In one study of men reporting persistent sexual symptoms after previous finasteride use, Basaria et al. (53) found no evidence of androgen deficiency or persistent inhibition of peripheral SRD5A gene activity, indicating that the pathophysiology of these complaints does not align with classical hypogonadism (53).

Available data regarding persistent adverse effects after discontinuation of dutasteride are very limited. In the observational cohort by Kiguradze et al. (54), persistent erectile dysfunction (PED) was reported in users of both finasteride and dutasteride, but cases linked to dutasteride represented only a small minority and were not analyzed separately due to low numbers (54). In controlled studies of dutasteride in men with AGA, sexual adverse events were generally mild to moderate and reversible. In the randomized 48-week trial, sexual adverse events occurred more often with dutasteride than placebo, but all resolved during treatment continuation or within weeks after discontinuation. No persistent or irreversible sexual dysfunction was observed in this AGA population (41). The observed regression of symptoms after treatment supports the hypothesis of their transient nature.

### 3.9 Female pattern hair loss

Female pattern hair loss (FPHL) has a substantial impact on quality of life. Topical minoxidil remains the first-line pharmacologic treatment; however, an insufficient or unsatisfactory clinical response is reported in a considerable proportion of patients (approximately 40%) (55). In selected cases, oral 5-ARIs have been used as off-label alternatives, and available clinical studies suggest potential efficacy in some subgroups of women (56). In published studies and clinical practice, finasteride doses in women generally range from 1 to 5 mg daily, with 2.5 mg daily being the most commonly reported, whereas dutasteride has been used at 0.5 mg daily (56). Because of the risk of teratogenic effects on the male fetus, 5-ARIs are contraindicated in pregnancy; in women of childbearing potential, pregnancy should be excluded before treatment and reliable contraception should be ensured throughout therapy (57).

In a study evaluating finasteride 5 mg daily for 18 months in 40 normoandrogenic postmenopausal women with AGA, Oliveira-Soares et al. (58) reported persistent decreased libido in 4 participants. Other reports of finasteride 5 mg have described occasional adverse events such as breast tenderness/swelling, menstrual irregularities, and increased body hair (59), whereas some studies did not observe or did not report such adverse effects (60–62). In a multicenter, randomized, double-blind, placebo-controlled trial including 137 women treated with finasteride 1 mg for 12 months, Price et al. (63) reported no sexual adverse effects in the finasteride group. The compiled findings suggest that adverse effects in women with androgenetic alopecia are rare or insufficiently reported, which may indicate a favorable safety profile.

### 3.10 The effect of 5-alpha reductase inhibitors on the development of breast cancer

Clinical data on cancer risk in 5-ARI users have been inconsistent. Meijer et al. (64) reported an increased incidence rate ratio of male breast cancer among finasteride users compared with non-users. In contrast, a large meta-analysis by Wang et al. (18), including 595,776 men, did not demonstrate a statistically significant association between 5-ARI use and male breast cancer risk (18). Additional real-world data have also suggested no clear association between dutasteride exposure and breast cancer (65).

In women with androgenetic alopecia, evidence remains more limited, but available cohort data do not suggest a clear increase in breast cancer risk with oral 5-ARIs. In a single-center retrospective cohort study by Venkatesh et al. (66) 810 women prescribed oral 5-ARIs (predominantly finasteride; a smaller proportion dutasteride) were compared with 5,472 matched controls, and no statistically significant difference in breast cancer risk was observed; similarly, no association was found with benign breast conditions (66).

Importantly, newer large-database evidence also addresses broader female reproductive cancer outcomes. Singal et al. (67) analyzed female patients with alopecia who were exposed to 5-ARIs, spironolactone, or minoxidil (control) between 2005 and 2025, using propensity-score matching to adjust for multiple relevant confounders. In matched analyses, exposure to 5-ARIs was not associated with increased risk of malignant or benign breast, uterine, or ovarian tumors compared with minoxidil-treated controls; median time from exposure to tumor diagnosis was also similar across groups for these outcomes (67). The compiled data indicate that potential signals of increased breast cancer risk are not supported by the majority of analyzed studies.

### 3.11 The influence of 5-alpha reductase inhibitors on the development of sexual dysfunction in benign prostatic hyperplasia

Benign prostatic hyperplasia (BPH) is a common condition in aging men and is a frequent cause of lower urinary tract symptoms (LUTS) (68). The disorder reflects nonmalignant enlargement of the prostate, with DHT playing a central role in prostatic growth and disease progression (69). Accordingly, 5-ARIs are widely used in BPH management; by lowering serum and intraprostatic DHT levels, they contribute to prostate volume reduction and symptom improvement over time (70).

In a meta-analysis, Liu et al. (15) evaluated the association between 5-ARI therapy and sexual adverse events across randomized trials in both BPH and AGA populations. In men treated for BPH, pooled relative risks suggested an increased risk of sexual dysfunction, erectile dysfunction, and decreased libido (15). In contrast, in trials conducted in AGA, the corresponding estimates were not statistically significant (sexual dysfunction: RR 1.21; 95% CI 0.85–1.72; erectile dysfunction: RR 0.66; 95% CI 0.20–2.25; decreased libido: RR 1.16; 95% CI 0.50–2.72) (15).

Other meta-analyses limited to BPH populations have reported less consistent findings. Jun et al. (71) found no significant increase in sexual dysfunction with finasteride or dutasteride (OR 0.83; 95% CI 0.64–1.08). Similarly, Li et al. (72) did not observe a significant difference in adverse events—including sexual adverse events—between dutasteride and finasteride in BPH treatment (72).

Table 6 provides a structured synthesis of sexual adverse event rates across all intervention groups identified in this review. Across placebo-controlled RCTs, event rates in treated patients were generally comparable to or only modestly higher than those in placebo groups, with consistent evidence of reversibility upon discontinuation. The highest rates of sexual adverse events were observed in observational studies of post-finasteride syndrome, though these findings derive from self-selected, uncontrolled populations and should be interpreted with caution. No sexual adverse events were reported in women treated with 5-ARIs for FPHL across any of the included studies.

**TABLE 6:** Summary of the study results.

	Treatment group	Number of studies	Number of patients	Sexual AE rate (treated)	Sexual AE rate (placebo/control)	Consistency of evidence	Reversibility
1	Oral Finasteride	11	7,116	0.25–7.9%	0.9–3.4%	High (multiple RCTs)	Mostly reversible
2	Oral Dutasteride	3	390	0–11.7%	0–5%	Moderate	Usually reversible
3	Topical Finasteride	4	167	0%	0%	High; no sexual side effects reported in any included study	N/A (no side effects reported)
4	Topical vs. Oral Finasteride (Direct Comparison)	3	532	Topical: 0–2.8% Oral: 0–4.8%	3.3% (for topical group)	High; topical consistently shows lower systemic impact.	Generally reversible
5	Oral Finasteride vs. Dutasteride (Direct Comparison)	4	2,023	Fin: 0.33–6.7% Dut: 0.44–13.0%	1.7–5.0%	Moderate; higher doses of Dutasteride correlate with higher AE.	Usually reversible.
6	Post-Finasteride Syndrome (PFS)	7	12,221 +	1.4% (PED) to 94% (Libido)	N/A	Controversial (self-reported data)	Persistent (May last for years)
7	Female pattern hair loss (FPHL)	6	376	0%	0%	Very high (no sexual AEs in women)	N/A

AE, Adverse events; PED, Persistent Erectile Dysfunction; N/A, Not applicable.

## 4 Discussion

5α-reductase inhibitors (5-ARIs) inhibit 5α-reductase, the enzyme that converts testosterone to the more potent androgen dihydrotestosterone (DHT) (7). Treatment with 5-ARIs reduces circulating DHT by approximately 70% while typically not altering circulating testosterone levels (15). Two major 5α-reductase isoenzymes have been described. Type I is expressed predominantly in the skin (including sebaceous glands and keratinocytes), whereas type II is highly expressed in androgen-responsive tissues of the male reproductive tract, including the prostate, seminal vesicles, epididymis, and vas deferens (16). Both isoenzymes contribute to DHT formation, with type II often considered the dominant source in androgen-sensitive reproductive tissues (16). Finasteride selectively inhibits type II 5α-reductase, whereas dutasteride inhibits both type I and type II isoenzymes (17). 5α-reductase is expressed in multiple tissues relevant to the clinical effects and adverse events of these drugs, including the prostate and scalp hair follicles (19). In scalp tissue, finasteride 5 mg/day has been reported to reduce DHT by approximately 41%, whereas dutasteride at a 10-fold lower dose has been reported to reduce scalp DHT by approximately 51%. In studies assessing the safety of dutasteride, the rate of sexual side effects was higher than with finasteride. Inhibition of both 5-alpha reductase isoenzymes may result in a more pronounced reduction in serum DHT levels. In the studies analyzed, dutasteride was associated with a higher rate of sexual side effects than finasteride. This difference may be related to dutasteride’s ability to inhibit both isoenzymes of 5-alpha-reductase, whereas finasteride acts selectively on only one of them. Consequently, serum DHT levels are reduced to a greater extent, which potentially increases the risk of symptoms such as decreased libido or erectile dysfunction.

The nocebo effect is a neurobiological phenomenon that can manifest as detectable changes in the body and lead to adverse health consequences (73). In randomized clinical trials, participants receiving a placebo often report side effects that are similar to those reported by patients receiving active treatment (74). It may be related to awareness of potential side effects during the informed consent process (75). Furthermore, these negative effects could occur due to negative expectations resulting from verbal suggestions, prior learning-based experiences, social observation, mass psychogenic modeling, negatively perceived patient-physician communication, and clinical encounters (73). In studies by Kaufman et al. (19), Kawashima et al. (22), Leyden et al. (23), Roberts et al. (30), Piraccini et al. (32), and Eun et al. (39) the incidence of sexual dysfunction in the placebo group was similar to that observed in patients receiving treatment. Patients’ awareness of potential side effects may significantly influence their subjective reporting of adverse events.

The following synthesis of post-finasteride syndrome (PFS) draws primarily on AGA-specific studies; observations from mixed-indication pharmacovigilance and cohort data are noted separately as supporting evidence where relevant. PFS is a term used to describe persistent adverse symptoms reported by some patients during finasteride therapy and/or after discontinuation of the drug (76). Reported manifestations most commonly involve sexual symptoms (e.g., decreased libido, erectile dysfunction, and ejaculatory disorders), but somatic complaints (e.g., fatigue, gynecomastia, and other physical symptoms) have also been described (77). In addition, finasteride use has been reported in association with other adverse outcomes, including male infertility, cataracts, intraoperative floppy iris syndrome, pseudoporphyria, and acute localized T-cell-mediated pustular eruptions; however, the strength and causality of these associations vary across reports (45). Studies by Giatti et al. (52) and Irwig et al. (78) have shown that the frequency and severity of post-finasteride syndrome symptoms may be greater than in patients currently undergoing treatment. However, the pathophysiology of this phenomenon remains unclear. Importantly, Basaria et al. (53) found no evidence of androgen deficiency or persistent inhibition of SRD5A gene activity in peripheral tissues; however, they noted differences in mood and brain activity patterns that correlated with sexual symptoms and negative affect. Moreover, while isolated reports of prolonged symptoms after dutasteride exist, the current evidence base—derived largely from mixed-indication cohorts including BPH patients (supporting evidence; Kiguradze et al.)—is small and does not establish a causal relationship or measurable incidence. In contrast to finasteride, documented cases are few, and in controlled AGA cohorts, sexual adverse effects appear predominantly reversible.

Evidence on the breast cancer risk associated with 5-ARI exposure derives from a heterogeneous mix of mixed-indication registry data and AGA-specific cohorts; the former are presented here as supporting evidence. Breast cancer in men is rare, with population-based incidence estimates on the order of approximately 1 per 100,000 person-years in the general population (64). Clinically, male breast cancer often presents similarly to breast cancer in postmenopausal women (18), most commonly as a palpable breast mass; less frequent presentations include nipple retraction, nipple discharge, ulceration of the nipple/skin, or Paget disease of the nipple (66). Reported survival outcomes in men are generally poorer than in women, which has been attributed to older age at diagnosis, more advanced stage at presentation, and lower awareness of the disease (67). Established risk factors include BRCA1/BRCA2 pathogenic variants, Klinefelter syndrome, states of androgen–estrogen imbalance, testicular disorders, obesity, and a history of prostate cancer and its treatment (79). From a mechanistic perspective, estrogens have been implicated in carcinogenesis through multiple pathways, including genotoxic and proliferative effects (80, 81). Because 5α-reductase inhibition reduces the conversion of testosterone to DHT, it may alter the androgen–estrogen balance, potentially increasing relative estrogenic exposure in some individuals (82, 83). The studies analyzed regarding breast cancer risk showed inconsistent results. Meijer et al. reported an increased risk of breast cancer, while Wang et al. did not confirm this association. Furthermore, in studies involving both retrospective single-center studies by Venkatesh et al. and large-database studies by Singal et al., no significant increase in breast cancer risk was demonstrated with the use of 5-ARIs. These findings provide reassurance regarding gynecologic and breast tumor risk in women treated with 5-ARIs for hair loss, while highlighting the need for long-term prospective studies with dose- and duration-stratified analyses.

Given that androgenetic alopecia is a chronic, largely elective indication for 5α-reductase inhibitors, clinicians should approach treatment as a shared decision in which expected hair outcomes are weighed against tolerability and patient priorities (2–4). Before initiating therapy, it is advisable to discuss both the typical, low-frequency sexual adverse events reported in randomized trials (e.g., decreased libido, erectile dysfunction, ejaculatory changes) and the uncertainty surrounding persistent symptom reports (often described as post-finasteride syndrome), emphasizing that causality and prevalence remain incompletely defined (39, 50, 51, 77, 84). Baseline assessment of sexual function, mood, and concomitant risk factors (including depressive symptoms, anxiety, relationship stressors, and use of other medications associated with sexual dysfunction) can help contextualize symptoms if they occur during follow-up. Patients should be informed that adverse effects, when present, are usually mild and may resolve with continued treatment or after discontinuation. They should also be encouraged to report symptoms early rather than stopping medication abruptly without medical guidance. If adverse effects emerge, clinicians may consider individualized strategies such as dose adjustment, switching to an alternative 5-ARI, or (where available) topical finasteride to reduce systemic exposure, alongside evaluation for other common causes of sexual dysfunction. For women, strict avoidance in pregnancy is essential, and treatment should be limited to carefully selected cases with documented counseling regarding teratogenic risk and the need for reliable contraception when applicable.

This systematic review has several limitations. First, a substantial proportion of records identified through the initial search (*n* = 8,320; 91% of 9,133 sought for retrieval) could not be obtained as full text, primarily reflecting the broad initial yield from Google Scholar (*n* = 20,734), which is enriched in gray literature, conference abstracts, preprints, dissertations, and records not indexed in subscription databases. Although our search covered four major databases and reference lists of relevant articles were manually screened, it is possible that some relevant studies were missed because of access restrictions. Second, the heterogeneity of study designs, populations, dosing regimens, and outcome definitions across included studies precluded a quantitative meta-analysis. Third, evidence on persistent sexual symptoms (post-finasteride syndrome) derives predominantly from self-selected, uncontrolled survey-based cohorts, which limits causal inference. Finally, available data in women with FPHL remain limited, and findings cannot be readily generalized to other female populations treated with 5-ARIs.

## 5 Conclusion

5-ARIs are effective therapies for androgenetic alopecia and are generally well tolerated. Across randomized trials in men treated for AGA, sexual adverse events (most commonly decreased libido, erectile dysfunction, and ejaculatory disorders) are typically uncommon and, in most reports, transient and reversible after discontinuation. However, observational and survey-based data indicate that a subset of patients may report persistent sexual symptoms after stopping finasteride (sometimes described as post-finasteride syndrome), although the prevalence, causal relationship, and underlying mechanisms remain uncertain and susceptible to reporting and selection bias. Among available 5-ARIs, topical finasteride may offer a favorable safety profile by reducing systemic exposure while maintaining clinical benefit, although longer-term comparative data are still needed. In women, oral 5-ARIs are used off-label in selected cases; available studies suggest potential efficacy, with inconsistent reporting of sexual or endocrine adverse effects, and strict pregnancy prevention is essential because of teratogenic risk. Overall, clinicians should counsel patients that most sexual side effects reported in controlled AGA studies are infrequent, mild, and reversible, but individual susceptibility varies. Shared decision-making, careful monitoring of sexual function, and further high-quality long-term studies—using standardized definitions of sexual dysfunction and persistence—are needed to quantify risks better and identify vulnerable subgroups.

## Data Availability

All data extracted and analyzed in this systematic review are included in the article. Further inquiries can be directed to the corresponding author.
